# Association Between the XRCC6 Polymorphisms and Cancer Risks: A Systematic Review and Meta-analysis

**DOI:** 10.1097/MD.0000000000000283

**Published:** 2015-01-09

**Authors:** Jing Jia, Juan Ren, Dongmei Yan, Long Xiao, Ruifen Sun

**Affiliations:** From the Center for Molecular Medicine, Zhejiang Academy of Medical Sciences, Hangzhou, Zhejiang 310013, P.R. China (JJ, JR, DY); Department of Urology, the First People's Hospital of Yunnan Province, KunMing University of Science and Technology, Kunming 650041, Yunnan, P.R. China (LX); Central Laboratory, Yunnan University of Chinese Traditional Medicine, Kunming 650500, Yunnan, P.R. China (RS); and Department of Immunology, West China School of Preclinical and Forensic Medicine, Sichuan University, Chengdu, Sichuan 610041, P.R. China (RS).

## Abstract

A number of studies have been carried out to investigate the association of X-ray repair complementing defective repair in Chinese hamster cells 6 (XRCC6) polymorphisms and cancer risks, and the results remained inconsistent and inconclusive.

To assess the effect of XRCC6 polymorphisms on cancer susceptibility, we conducted a meta-analysis, up to May 23rd 2014, 6267 cases with different types of tumor and 7536 controls from 20 published case–control studies. Summary odds ratios and corresponding 95% confidence intervals for XRCC6 polymorphism and cancer risk were estimated using fixed- or random-effects models when appropriate. Heterogeneity was assessed by chi-squared-based Q-statistic test, and the sources of heterogeneity were explored by subgroup analyses, logistic meta-regression analyses and Galbraith plot. Publication bias was evaluated by Begg funnel plot and Egger test. Sensitivity analyses were also performed.

The rs2267437 polymorphism was associated with a significant increase in risks of overall cancers, breast cancer, renal cell carcinoma and hepatocellular carcinoma, and it could increase the cancer risk in Asian population; the rs5751129 polymorphism could increase the cancer risk in overall cancers; the rs132770 polymorphism was associated with the increased renal cell carcinoma risk; furthermore, the rs132793 polymorphism could decrease breast cancer risk and increase risks in “other cancers”.

Overall, the results provided evidences that the single nucleotide polymorphisms in XRCC6 promoter region might play different roles in various cancers, indicating different cancers have different tumorigenesis mechanisms. Our studies may perhaps supplement for the disease monitoring of cancers in the future, and additional studies to determine the exact molecular mechanism might provide us with interventions to protect the susceptible subgroups.

## INTRODUCTION

Cancer is a major public health problem in the world, and it is among the leading causes of death over world. In the year of 2014, there will be 1,665,540 new cancer cases and 585,720 cancer deaths expected to occur; of these, 51.3% of the cases and 52.9% of the deaths occurred in males.^1^ Cancer survival tends to be poor, and most cancers are diagnosed at a late stage and the limited efficient treatments due to the unclear cancer pathogenesis mechanisms.

Genomic instability is one cause of carcinogenesis in human cancers, which could promote various mutations. DNA double-strand break (DSB) is the most serious type of damage, and the unpaired or incorrectly repaired DSB could lead to genomic instability.^2,3^ In mammalian cells, DSB can be repaired mainly by non-homologous end joining (NHEJ) pathway, involving several DSB repair genes, such as X-ray repair complementing defective repair in Chinese hamster cells 6 (*XRCC6*).^4^ Genetic variations in NHEJ genes, such as single nucleotide polymorphism (SNP) might escape cell checkpoint surveillance, and can lead to suboptimal DNA repair, allowing DNA damage to accumulate, and finally trigger tumor initiation.^5–7^

*XRCC6* is a gene coding Ku70 protein. *XRCC6* gene is one component of NHEJ pathway. It plays an important role in suppression of chromosomal rearrangements and maintenance of genome integrity, thus it is considered essential for genome stability and cell survival. Genetic polymorphism in the *XRCC6* gene is hypothesized to have a critical role in tumorigenesis. A number of studies have been carried out to investigate the association of *XRCC6* polymorphisms and cancer risks, such as breast cancer,^8,9^ lung cancer,^10^ hepatocellular carcinoma (HCC),^11,12^ glioma,^13^ and so on, however, the results are inconsistent and inconclusive. Also, several functional studies have been carried out to demonstrate the effects of *XRCC6* polymorphisms on Ku70 transcriptional activity in limited types of cancers.^14–16^ The overall influence of *XRCC6* polymorphisms on cancer risks is still ambiguous.

Until recently, the meta-analyzes assessing the association between *XRCC6* polymorphisms and cancer^17–19^ are limited to one single polymorphism site. Systematic review of association between *XRCC6* polymorphisms and cancer risk is lacking, and the role of *XRCC6* in the etiology of cancer is still equivocal.

We carried out a meta-analysis on all updated published case–control studies to estimate the overall tumor risk of *XRCC6* polymorphisms in Asian and European population and to investigate heterogeneity between the individual studies as well as the existence of potential publication bias. The results provided evidences that the SNPs in *XRCC6* promoter region might associate with the cancer risks, while SNPs in the *XRCC6* intron might not.

## MATERIALS AND METHODS

### Selection of Published Studies

Studies dealing with the association between cancer risk and *XRCC6* gene polymorphisms were considered eligible. We searched the PubMed, Web of Science and Embase databases for all articles on the association between *XRCC6* polymorphism and cancer risk (last search update May 23rd 2014). The following terms were used in this search: “Ku70, *XRCC6* or X-ray repair complementing defective repair in Chinese hamster cells 6” and “cancer, carcinoma or tumor” and “polymorphism or polymorphisms”. Additional eligible studies on this topic were identified by a hand search of references of retrieved articles. Studies testing the association between *XRCC6* gene polymorphism and cancer were included if all the following conditions were met^5,8–14,16,20–30^: the publication was a case–control study; the study provided the total number of cases and controls; the study provided available genotype frequency in case and control group, respectively; the study was published in English. The major exclusion criteria were as follows: duplicate data^31^; abstract, comment, review or editorial.^17–19^ Finally, 20 case–control studies including 6267 cancer cases and 7536 cancer-free controls were included in this meta-analysis, studying the association between different *XRCC6* polymorphisms with various cancer risks in Asian and European population. The publication year of eligible studies ranged from 2003 to 2014. Among them, 17 studies concerning *XRCC6* C1310G (rs2267437) polymorphism (case/control: 5061/6406), 7 studies concerning T-991C (rs5751129) polymorphism (case/control: 1875/3133), 9 studies concerning A-31G (rs132770) polymorphism (case/control: 4768/3517), 6 studies concerning intron 3 (rs132774) polymorphism (case/control: 1468/2738), 4 studies concerning A46922G (rs132793) polymorphism (case/control: 1824/1807), respectively. Polymorphisms of rs132778, rs12163239, and rs6519265 with only 1 concerned article were excluded.

No contacts with authors were carried out. Ethical approval and informed patient consent was not required as this study was a literature review and had no direct patient contact or influence on patient care.

### Data Extraction

Two investigators (Ren and Yan) independently extracted data from each study with a predefined review form, and discrepancies were resolved by consensus of all investigators. All study personnel were blinded throughout the meta-analysis. The following information was recorded for each study: the surname of first author, year of publication, cancer type, ethnicity, number of cases and controls, genotyping methods, matching variables, minor allele frequency in controls, and status of Hardy–Weinberg equilibrium (HWE).

To assess the quality of each eligible case–control study, the same two investigators (Ren and Yan) worked independently to determine the adequacy of studies selection, and discrepancies resolved by discussion with all investigators. All assessors were blinded throughout the meta-analysis. The followings were assessed: the cases and control definition; the comparability of cases and controls on the basis of the design or analysis; the genotyping examinations of polymorphisms; the ascertainment methods for cases and controls; the sources of control; and HWE status of controls.

Cases and controls were marched in all the 20 studies. Among them, 12 studies provided age- gender-marched controls, 5 studies provided additional factor marched controls besides age and gender, such as ethnic background, residence area, individual habits, and 3 studies provided gender-marched controls (Tables 1–5).

**TABLE 1:**
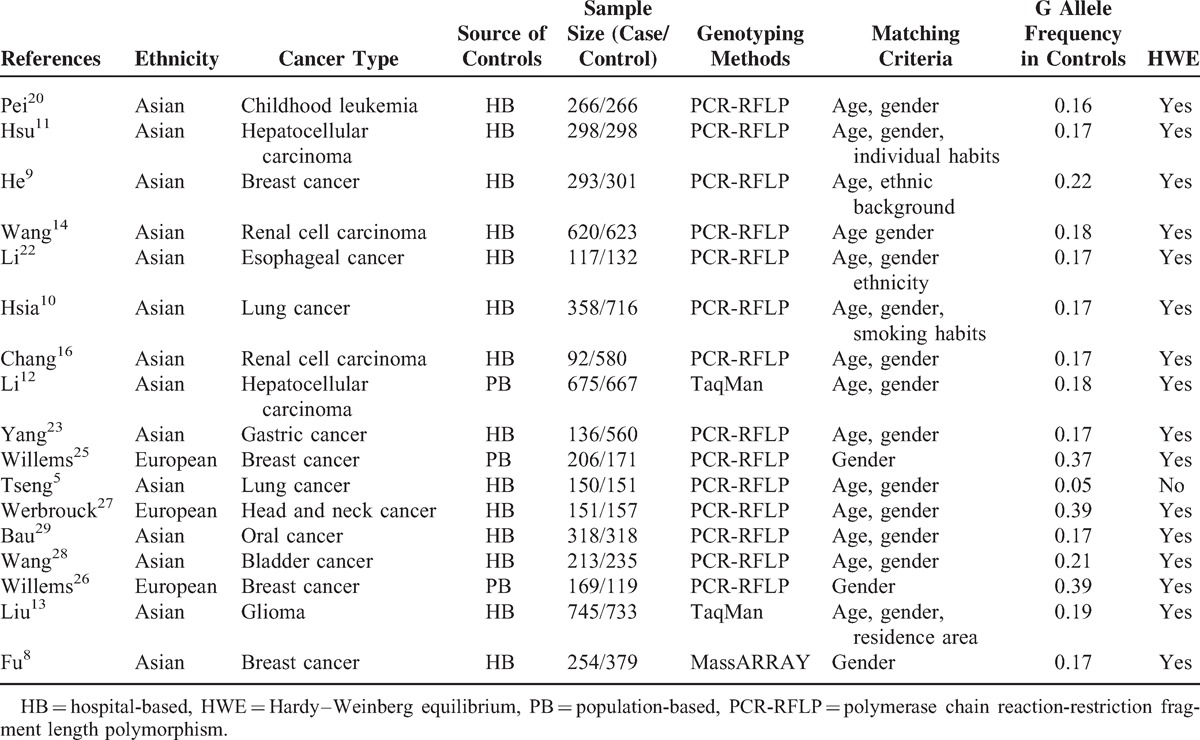
Characteristics of Literatures on XRCC6 rs2267437 Polymorphism Included in the Meta-Analysis

**TABLE 2:**
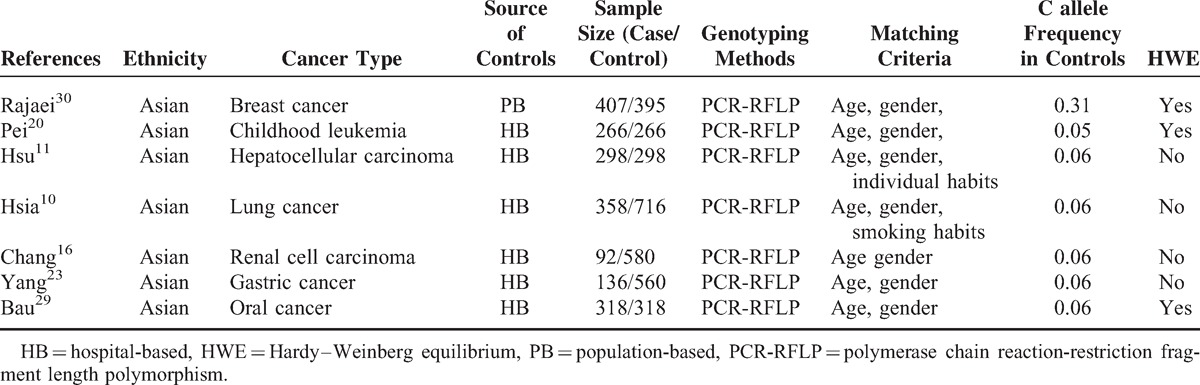
Characteristics of Literatures on XRCC6 rs5751129 Polymorphism Included in the Meta-Analysis

**TABLE 3:**
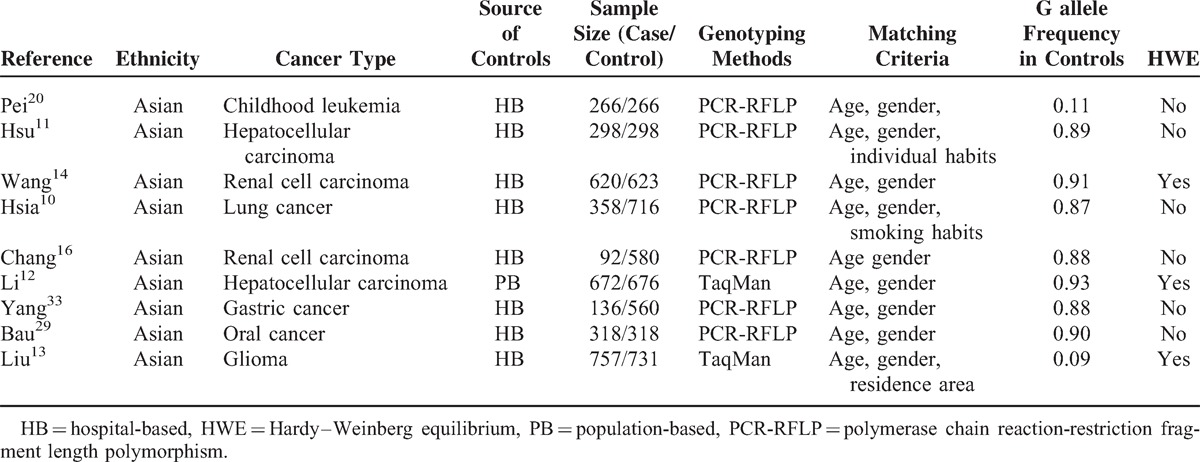
Characteristics of Literatures on XRCC6 rs132770 Polymorphism Included in the Meta-Analysis

**TABLE 4:**
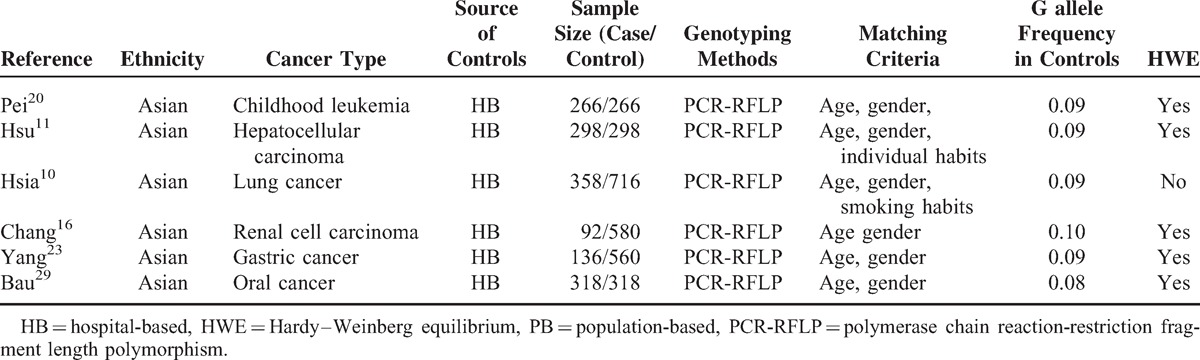
Characteristics of Literatures on XRCC6 rs132774 Polymorphism Included in the Meta-Analysis

**TABLE 5:**
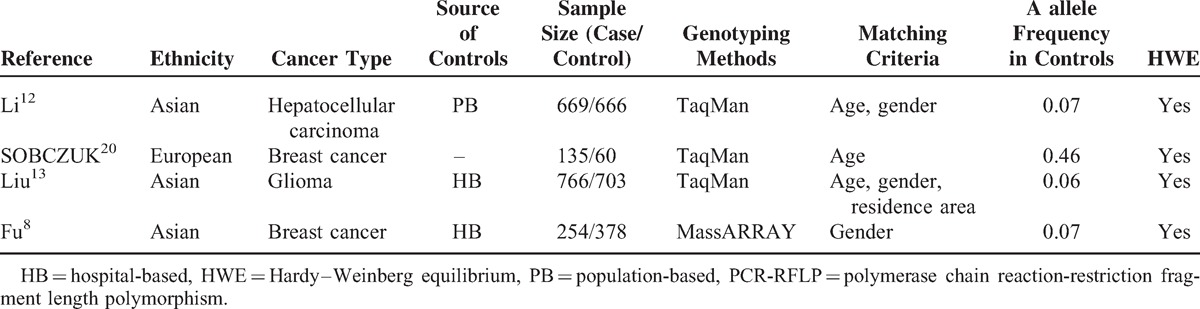
Characteristics of Literatures on XRCC6 rs132793 Polymorphism Included in the Meta-Analysis

### Statistical Analysis

Deviation from HWE in the control group was examined by χ^2^ test, *P* < 0.05 was considered to be statistically significant.^32^ The strength of relationship association between *XRCC6* polymorphisms and cancer risk was assessed by using the pooled odds ratio (OR) with 95% confidence intervals (95% CI). The significance of the pooled OR was determined by Z test, with *P* < 0.05 considered statistically significant. We evaluated the risk using the allele model (A vs. a), the homozygous model (AA vs. aa), the heterogeneity model (Aa vs. aa), the dominant model (AA + Aa vs. aa), and the recessive model (AA vs. Aa + aa). The chi-squared-based Q-statistic test was used to assess heterogeneity. When the result of the heterogeneity test was *P* < 0.05, the random-effects model was used (the DerSimonian and Laird method).^33^ Otherwise, the fixed-effects model was selected (the Mantel–Haenszel method).^34^ To explore sources of heterogeneity across studies, we did subgroup analyses and logistic meta-regression analyses.^35^ Galbraith plot was also used to explore sources of heterogeneity when meta-regression analyses could not find the heterogeneity source.^36^ Subgroup analysis based on ethnicity, cancer types (if one cancer type contains only one study, it was merged into the “other cancers” group), source of controls (hospital-based studies and population-based studies), HWE status of controls (HWE consistent: *P* > 0.05 and HWE inconsistent: *P* < 0.05) was performed. Funnel plots and Egger linear regression were used to diagnose a potential publication bias.^37^ For the possible publication bias, trim and fill method was used to evaluate the influence to the result.^38^ Sensitivity analyses were performed to assess the stability of the results by excluding one study at a time. All analyses were done, using STATA software, version 10.0 (STATA Corp., College Station, TX, USA). All graphs were obtained also by STATA software. All the *P* values were two-sided. Data from this meta-analysis are presented in accordance with the checklist proposed by the Meta-Analysis of Observational Studies in Epidemiology group.^39^

## RESULTS

### Characteristics of Studies

Overall, 20 studies including 6267 cases and 7536 controls, concerning 5 *XRCC6* polymorphisms were included in this meta-analysis. Study characteristics are summarized in Tables 1, 2, 3, 4 and 5.

The 20 articles concerned *XRCC6* C1310G (rs2267437) polymorphism, T-991C (rs5751129) polymorphism, A-31G (rs132770) polymorphism, intron 3 (rs132774) polymorphism, A46922G (rs132793) polymorphism, respectively. Among the 20 studies, 6 investigated breast cancer, 3 investigated renal cell carcinoma (RCC), 2 investigated HCC, 2 investigated lung cancer, and 1 for oral cancer, childhood leukemia, esophageal cancer, gastric cancer, bladder cancer, glioma, head and neck cancer each. There were 4 studies of European descendents, 16 studies of Asian descendents, among which 15 studies were of Chinese population. For the selection of controls, 4 studies were population-based case–control studies, while the remaining 16 were hospital-based case–control studies.

Several genotyping methods were used, including Taq-Man, polymerase chain reaction-restriction fragment length polymorphism (PCR-RFLP), MassARRAY. 60% (12/20) of these studies included described genotyping quality control measures, such as positive and negative controls, blindness to the case–control status, a different genotyping assay to confirm the data, and/or random repetition of a portion of samples.

### Association Between the XRCC6 Polymorphisms and Cancer Risk

**FIGURE 1:**
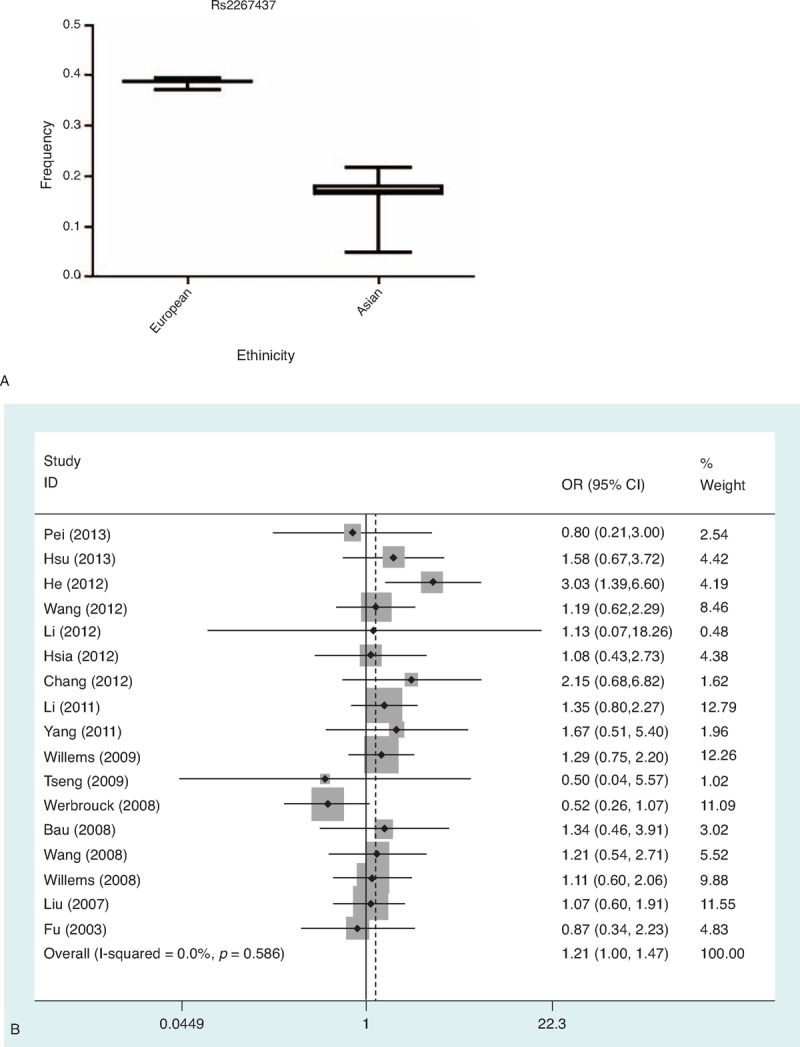
A. Frequencies of the *XRCC6* rs2267437 G allele among control subjects stratified by ethnicity. B. Forest plot of cancer risk associated with the GG genotypes compared with the CC/CG genotype in *XRCC6* rs2267437 polymorphism.

**FIGURE 2:**
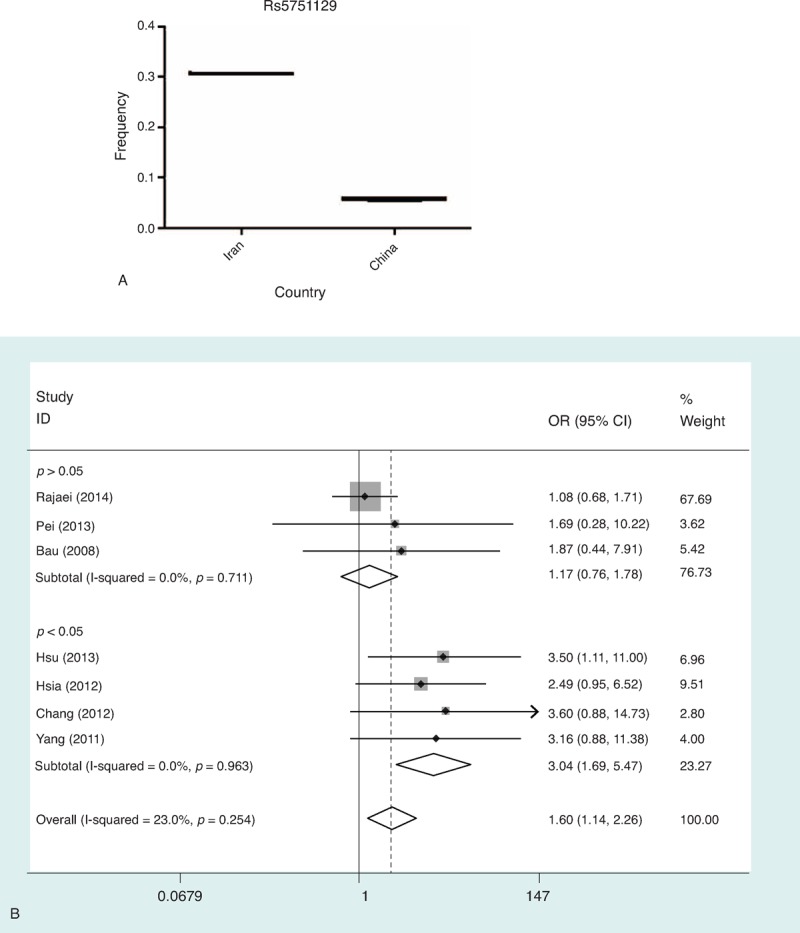
A. Frequencies of the *XRCC6* rs5751129T/C among control subjects stratified by nation. B. Forest plot of cancer risk associated with the CC genotypes compared with the TT genotype in *XRCC6* rs5751129 polymorphism in subgroup analysis of HWE status.

The G allele frequency in rs2267437 polymorphism varied widely between Asian and European ethnicities, ranging from 0.05 in an Asian population to 0.39 in a European Population. The mean frequency of G allele was 0.17 for Asian, and 0.38 for European (Figure 1A, Table 1). The C allele or G allele frequency in rs5751129 polymorphism or rs132793 polymorphism also varied widely in different populations (Figures 2A and 4A).

In overall comparison, there was obvious evidence of an association between *XRCC6* rs2267437 polymorphism and increased cancer risk (GG vs. CC: OR = 1.33; 95% confidence intervals (CI), 1.09–1.62; recessive model (GG vs. CC/CG): OR = 1.21; 95% CI 1.00–1.47) (Table 6  and Figure 1B). There was also obvious evidence of an association between *XRCC6* rs5751129 polymorphism and increased cancer risk (C vs. T: OR = 1.93, 95% CI 1.42–2.63; CC vs. TT: OR = 1.60; 95% CI, 1.14–2.26; TC vs. TT: OR = 1.95, 95% CI 1.48–2.57; dominant model: OR = 2.00, 95% CI 1.50–2.67; recessive model: OR = 1.48, 95% CI 1.06–2.06) (Table 7).

**TABLE 6:**
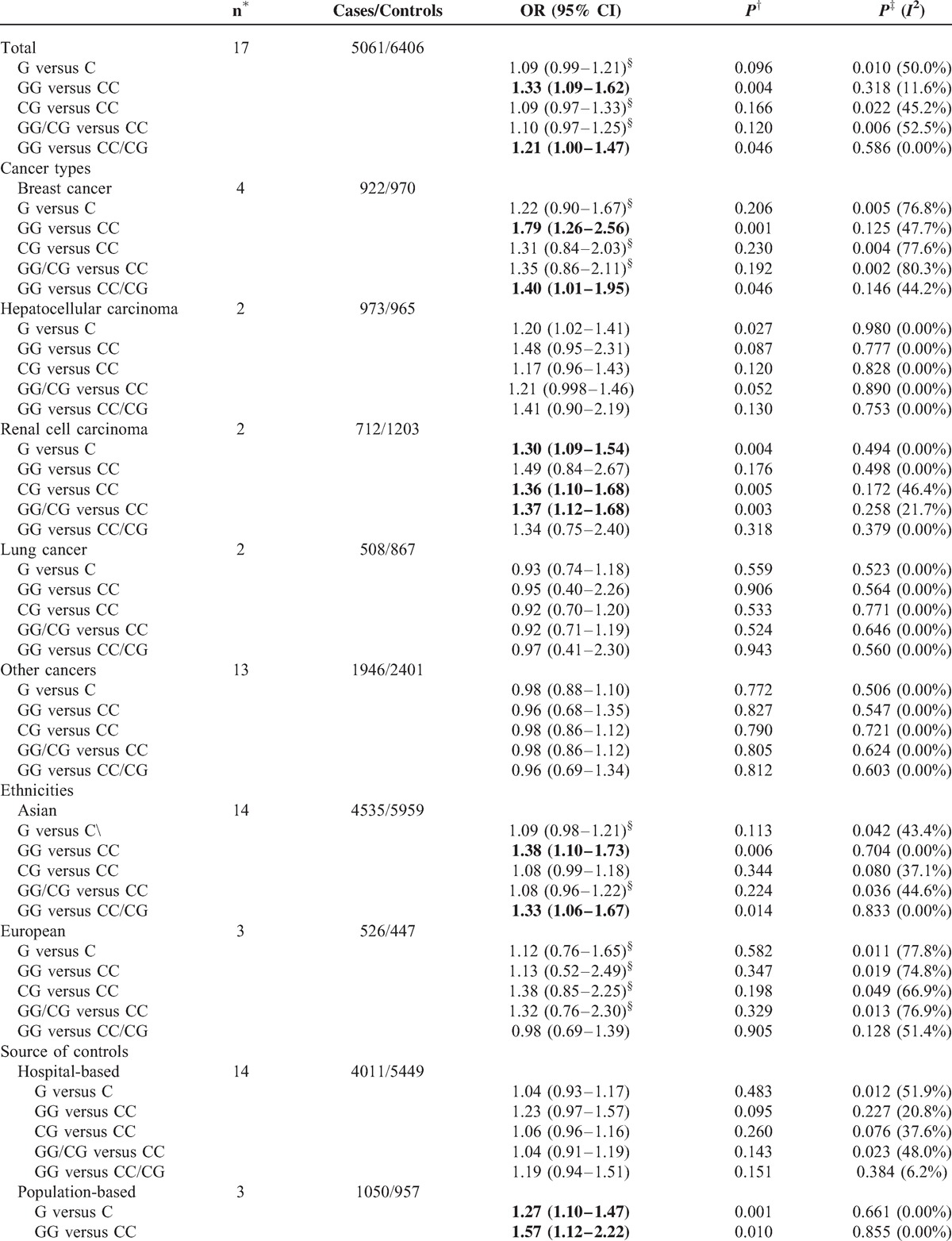
Stratified Analyses of the XRCC6 rs2267437 Polymorphism on Cancer Risk

**TABLE 6 (Continued):**
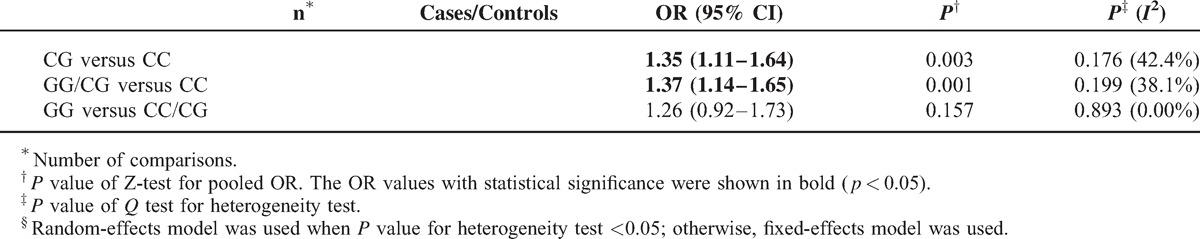
Stratified Analyses of the XRCC6 rs2267437 Polymorphism on Cancer Risk

**TABLE 7:**
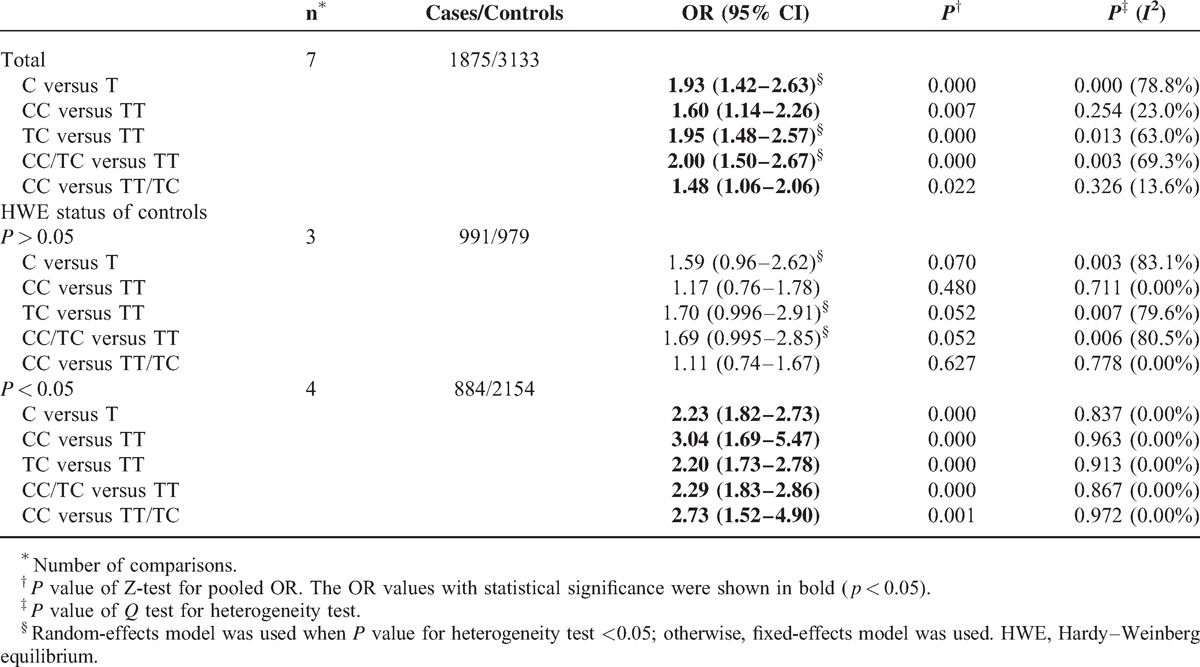
Stratified Analyses of the XRCC6 rs5751129 Polymorphism on Cancer Risk

**FIGURE 3:**
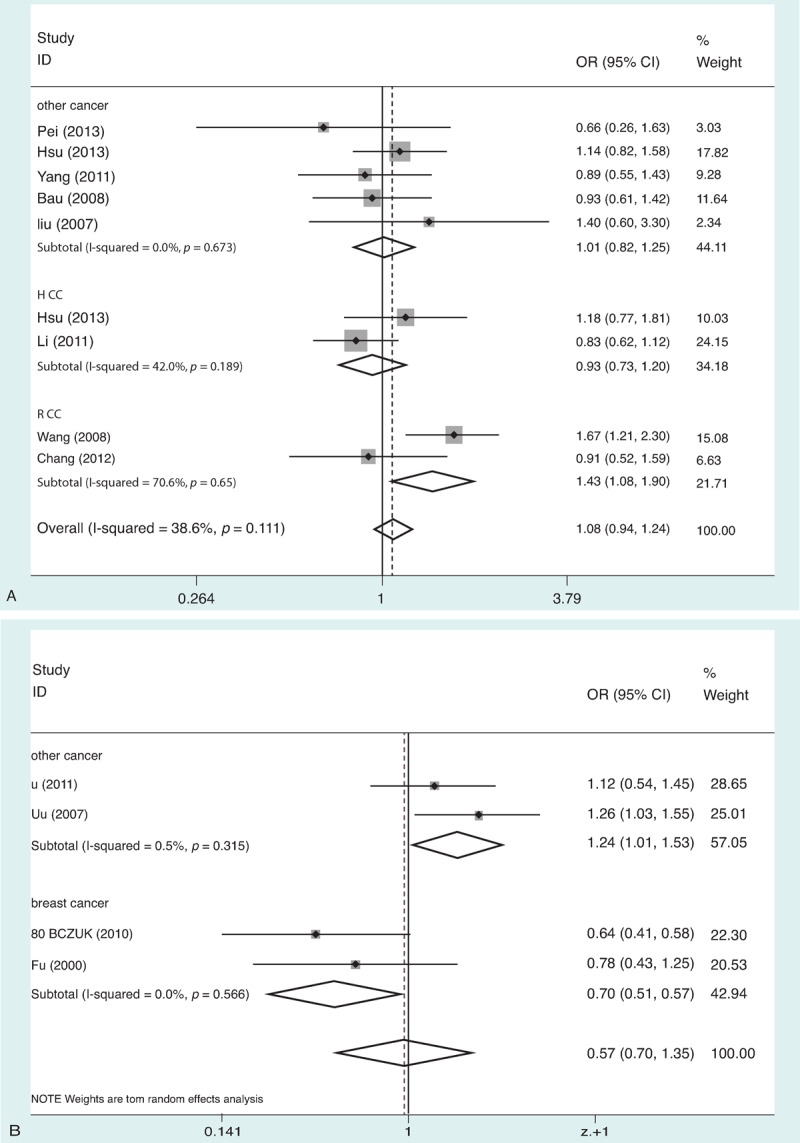
A. Forest plot of cancer risk associated *XRCC6* rs132770 polymorphism in subgroup analysis of cancer type in recessive model. B. Forest plot of cancer risk associated with the A genotypes compared with the G genotype in *XRCC6* rs132793 polymorphism in subgroup analysis of cancer type.

In subgroup analysis, polymorphism rs2267437 was found to be associated with increased cancer risk in breast cancer (GG vs. CC: OR = 1.79, 95% CI 1.26–2.56; recessive model: OR = 1.40, 95% CI 1.01–1.95), HCC (G vs. C: OR = 1.20, 95% CI 1.02–1.41), and RCC (G vs. C: OR = 1.30, 95% CI 1.09–1.54; CG vs. CC: OR = 1.36, 95% CI 1.10–1.68; dominant model: OR = 1.37, 95% CI 1.12–1.68). Similarly, significantly increased risks were observed in the Asian population (GG vs. CC: OR = 1.38, 95% CI 1.10–1.73; recessive model: OR = 1.33, 95% CI 1.06–1.67) in rs2267437 polymorphism (Table 7). The rs132770 polymorphism might associate with increased RCC risk (G vs. A: OR = 1.40, 95% CI 1.08–1.80; recessive model: OR = 1.44, 95% CI 1.08–1.90) (Table 8, Figure 3A). Interestingly, significantly decreased risks were observed in breast cancer in rs132793 polymorphism (A vs. G: OR = 0.70, 95% CI 0.51–0.97), however increased risks were found in “other cancers” in the same polymorphism (A versus G: OR = 1.24, 95% CI 1.01–1.53; GA vs. GG: OR = 1.29, 95% CI 1.03–1.60; dominant model: OR = 1.28, 95% CI 1.03–1.58) (Table 10 and Figure 3B).

**TABLE 8:**
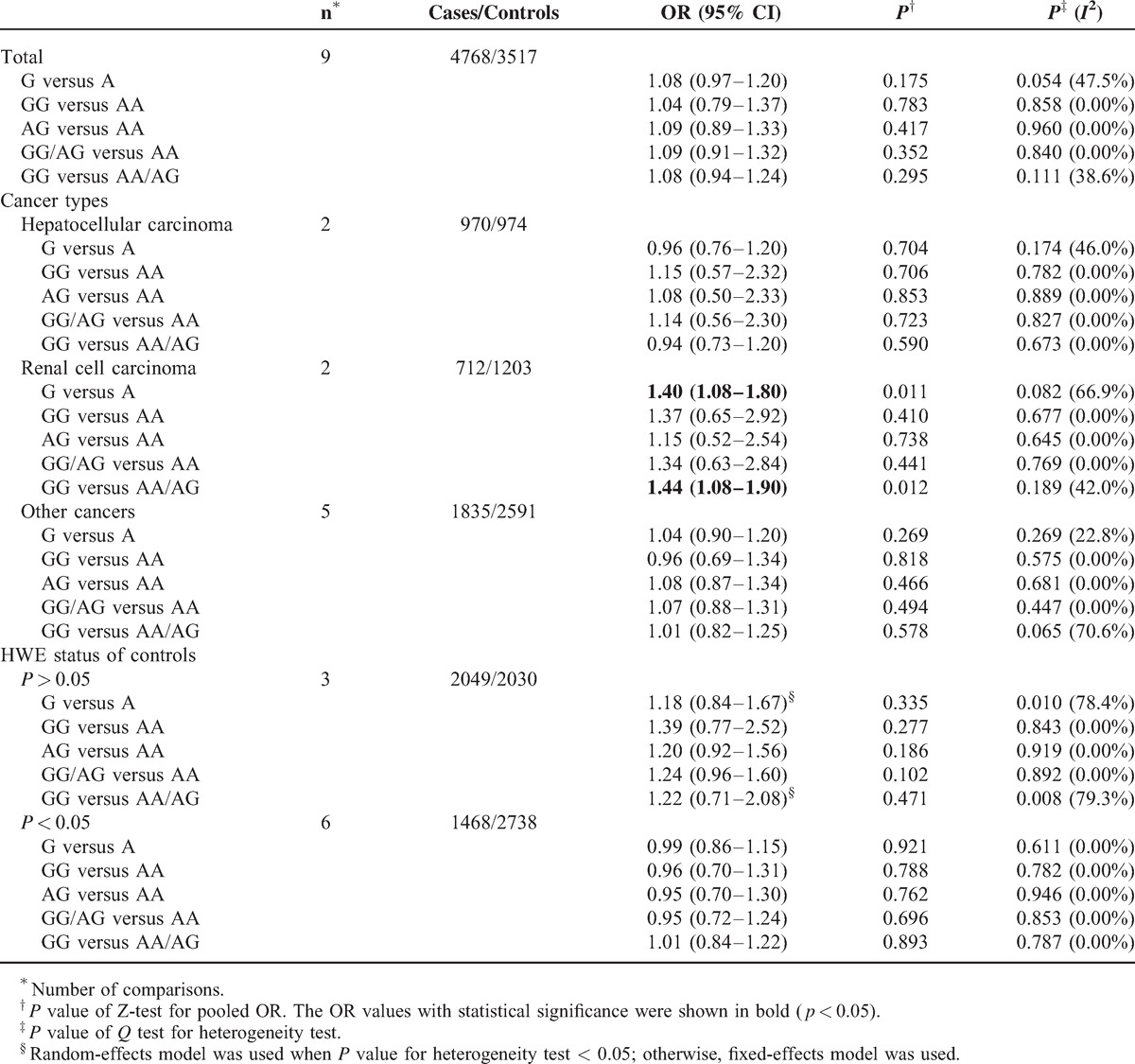
Stratified Analyses of the XRCC6 rs132770 Polymorphism on Cancer Risk

In addition, when stratified by HWE status of controls. Significantly elevated risks were observed in HWE inconsistent studies of rs5751129 polymorphism (C vs. T: OR = 2.23, 95% CI 1.82–2.73; CC vs. TT: OR = 3.04; 95% CI, 1.69–5.47; TC vs. TT: OR = 2.20, 95% CI 1.73–2.78; dominant model: OR = 2.29, 95% CI 1.83–2.86; recessive model: OR = 2.73, 95% CI 1.52–4.90) (Table 7, Figure 2B). No associations were found in HWE consistent studies.

After stratified separately by “sources of control”, significantly elevated risk was found in population-based studies in all comparison models tested except for recessive model of rs2267437 polymorphism (G vs. C: OR = 1.27, 95% CI 1.10–1.47; GG vs. CC: OR = 1.57, 95% CI 1.12–2.22; CG vs. CC: OR = 1.35, 95% CI 1.11–1.64; dominant model: OR = 1.37 95% CI 1.14–1.65) (Table 7).

No association was found between the *XRCC6* rs132774 polymorphism and cancer risk (Table 9).

**TABLE 9:**
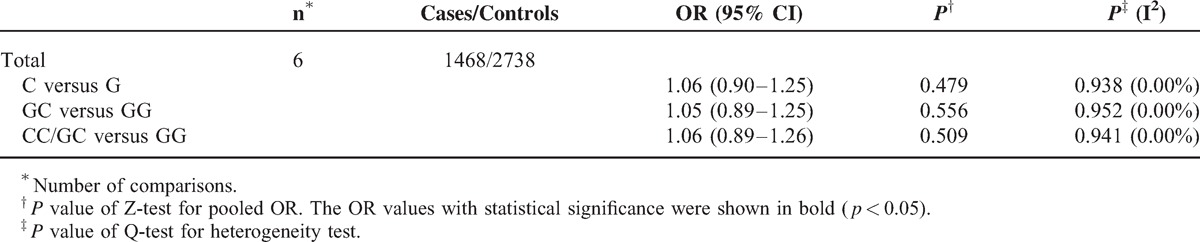
Stratified Analyses of the XRCC6 rs132774 Polymorphism on Cancer Risk

**TABLE 10:**
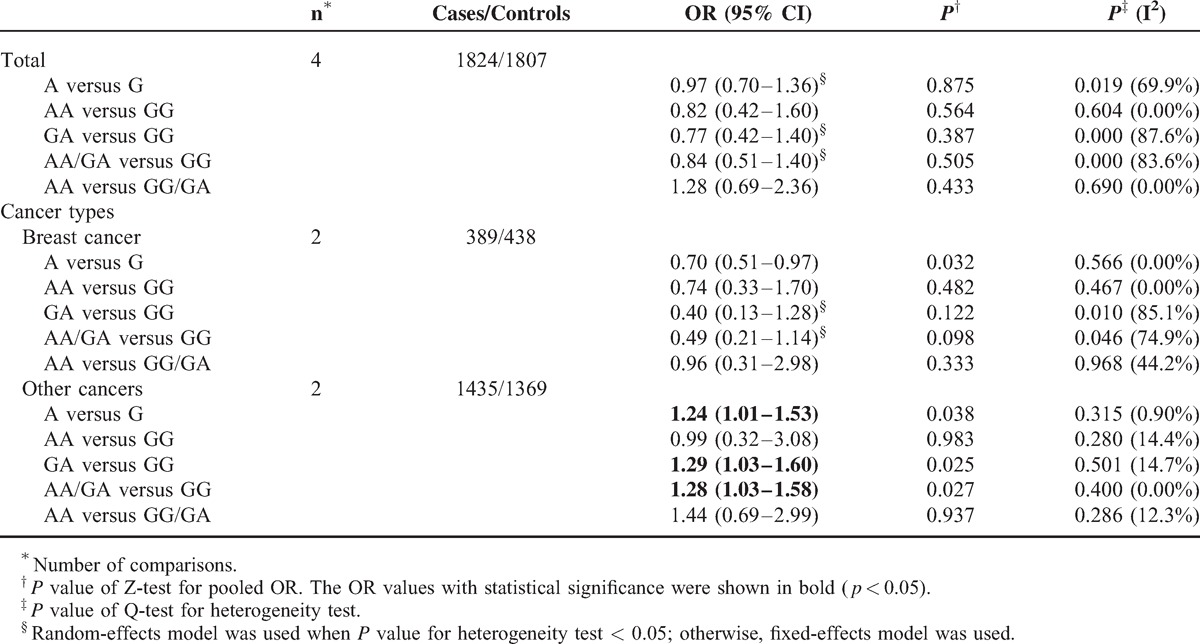
Stratified Analyses of the XRCC6 rs132793 Polymorphism on Cancer Risk

### Evaluation of Heterogeneity

There was heterogeneity among studies in overall comparisons and also subgroup analyses in 3 *XRCC6* polymorphisms: rs2267437, rs5751129, and rs132793. To explore sources of heterogeneity, we evaluated the following variables: ethnicities, cancer type, source of control, study quality, genotyping methods and sample size (≤500 and >500 subjects). Galbraith plot was also used to detect the possible sources of heterogeneity when none of the above variables could explain the heterogeneity.

**FIGURE 4:**
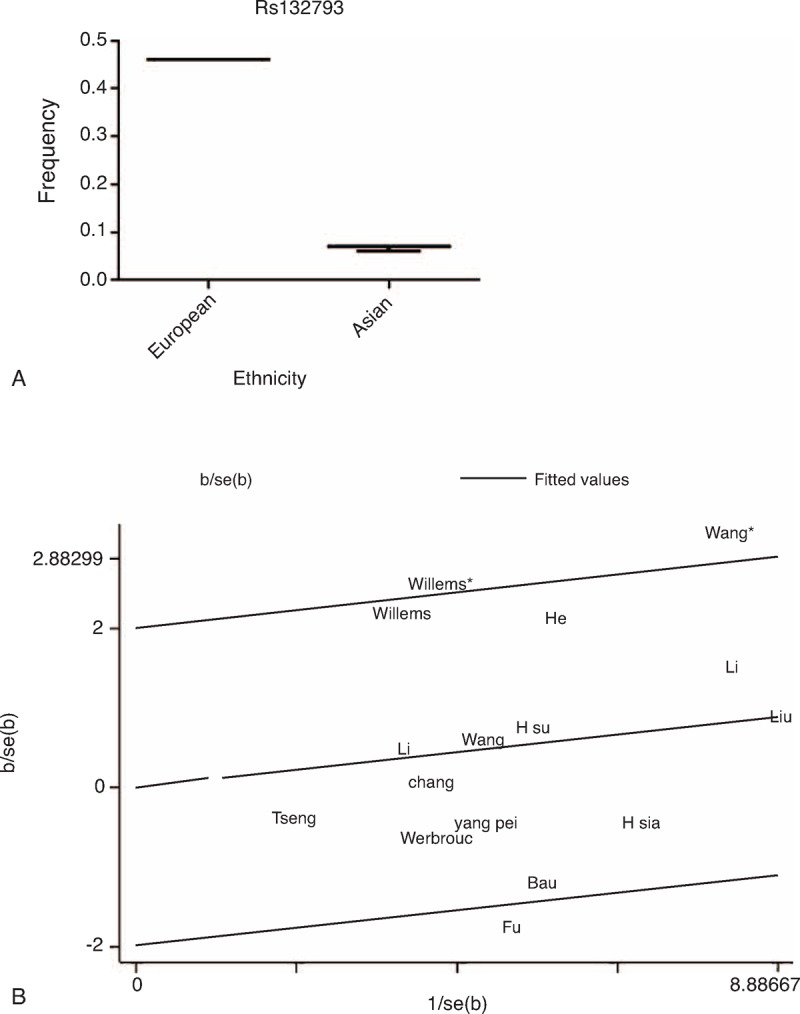
A. Frequencies of the *XRCC6* rs132793 among control subjects stratified by country. B Identification of studies acting as sources of heterogeneity by the Galbraith plot under the heterozygous model in rs2267437 polymorphism. Each name represents a separate study for the indicated association. The random effects model was used. Wang^∗^: ^14^, Willems^∗^: ^25^.

For the rs2267437 polymorphism, there was significant heterogeneity in overall comparisons of allele model, heterozygote model, and dominant model. None of the possible variables could explain the heterogeneity. The studies potentially causing between-study heterogeneity were identified in the allele model^8,9,27^), heterozygote model^8,14,25^, and dominant model^8,9,14,25^ by the Galbraith plot (Figure 4B). However, the result was altered in allele model (OR = 1.13 95% CI 1.05–1.21) when the studies were excluded.

Meta-regression analysis indicated that the “source of control” could explain 100% of the τ^2^ in all comparison models in rs5751129 polymorphism, and the “cancer type” could explain 100% of the τ^2^ in allele model in rs132793 polymorphism. Furthermore, the combination of “ethnicity” and “cancer type” could explain 100% of the τ^2^ in heterozygote and dominant model in rs132793 polymorphism.

### Sensitivity Analysis

**FIGURE 5:**
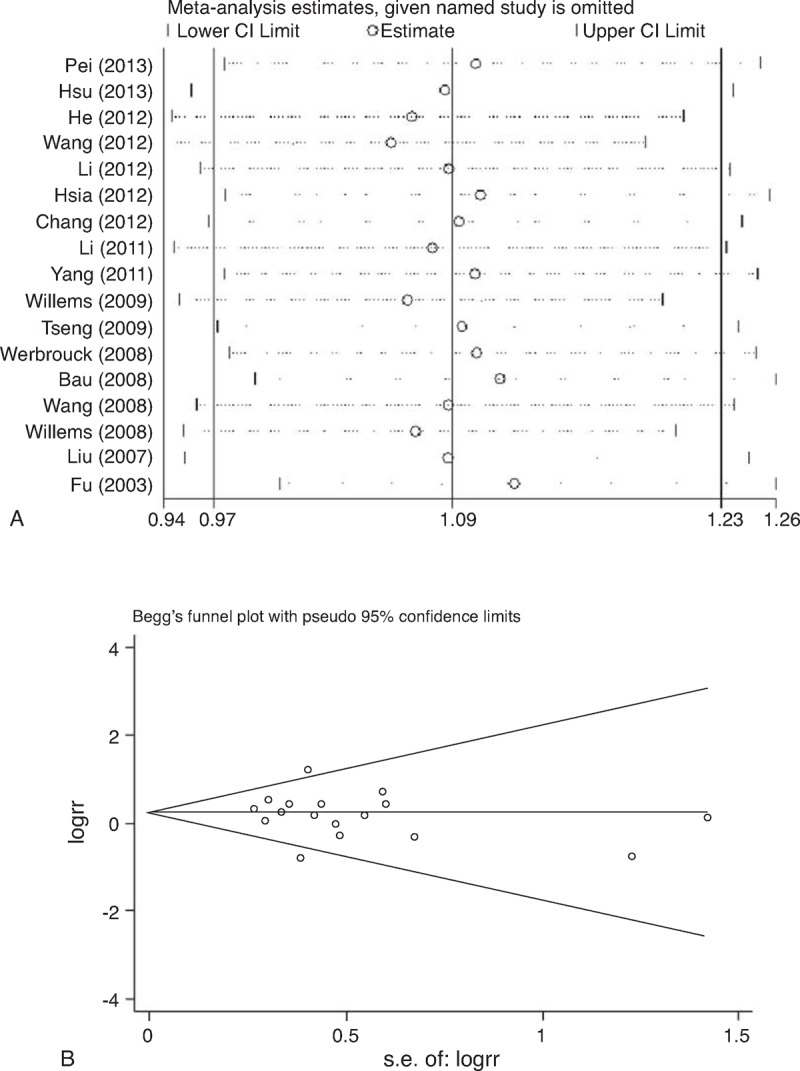
A. Sensitivity analysis of the heterozygous model in *XRCC6* rs2267437 polymorphism. B. Begg funnel plot for publication bias test under homozygous model in rs2267437 polymorphism. Each point represents a separate study for the indicated association.

Sensitivity analyses were performed by sequential removal of each eligible study to assess the influence of each individual study on the pooled OR in each comparison in the polymorphisms of rs2267437, rs5751129, and rs132793. The omission of any study made no significant difference, indicating that the results of this meta-analysis were statistically reliable (Figure 5A).

### Publication Bias

**FIGURE 6:**
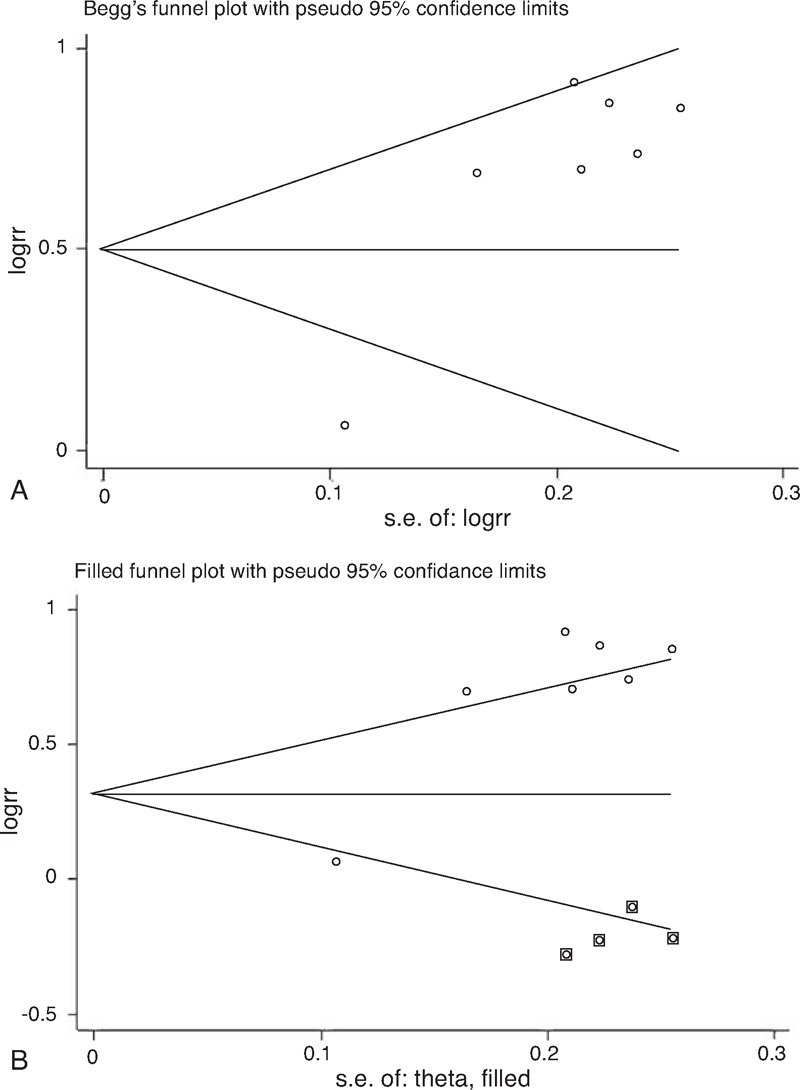
Begg Funnel Plots of Meta-Analysis before (A) and after (B) Trim and Fill Adjustment for Publication Bias under allele model in *XRCC6* rs5751129 polymorphism.

Egger test reveals evidence of publication bias in rs5751129 polymorphism (Figure 5B). The trim and fill method showed that the funnel plot needs 4 more studies to be symmetrical (Figure 6) in allele model, recessive model and homozygous model, or 2 more in heterozygous and dominant model. But the results were altered in recessive model and homozygous model. The publication bias in rs132793 polymorphism was not checked because of the limited number of relevant studies.

## DISCUSSION

In this study, we performed a systematic review of association between *XRCC6* polymorphisms and cancer risks based on 20 case–control studies. This is also the first time to explore the individual association between the polymorphisms of rs5751129, rs132774, rs132793, and cancer risk. The results provided evidences that the SNPs in *XRCC6* promoter region might associate with the cancer risks, while SNP in the *XRCC6* intron might not. In addition, the *XRCC6* promoter SNPs might play different roles in various cancers, indicating that *XRCC6* gene may have different roles in different cancer development, and different mechanisms promote the development of various tumors.

*XRCC6* gene was involved in multiple cellular pathways, including NHEJ pathway of DSB repair.^40,41^ During DNA repair procedure, Ku70 acted as a scaffold to recruit the other NHEJ factors to the damage site after its bound to DNA ends.^4^ However, inaccurate repair could lead to cellular aberrant function, apoptosis, and chromosomal rearrangements, which promote carcinogenesis finally.^42^ Besides the influence of *XRCC6* gene on DSB repair and genomic stability, it has some other NHEJ-independent effects. Such as the Ku70 protein in cytoplasm can prevent Bax translocate to mitochondria, and suppresses cell apoptosis.^43^ The defects in Ku70 may also influence cell proliferation.^44^

It has been demonstrated that the rs2267437 polymorphism could influence the expression level and stability of the Ku70 protein in breast cancer cells and RCC tissues.^14,15^ The sequence variation in the rs2267437 may affect binding activity of the adjacent CACCC box with transcription factors, resulting in decreased Ku70 expression level, and the DSB repair activity thus was affected, finally leading to increased susceptibility to cancers.^45^ In our study, rs2267437 polymorphism was found to might increase the cancer risks in breast cancer, HCC and RCC, while may not function in lung cancer or some other cancers, and different ethnicities might influence the association.

The rs5751129 polymorphism locates between rs132770 and rs2267437 in the promoter region of *XRCC6* gene. This polymorphism was proved to be functional in HCC and RCC, with the normal tissues with C allele having a lower expression level of *XRCC6* mRNA or protein.^11,16^ Our results indicated that rs5751129 polymorphism might play the same role in various cancers. The variation may influence the expression level or stability of *XRCC6* mRNA via alternative spicing or other mechanisms. Individuals carrying C allele may exhibit heritable decreased DNA repair capacity phenotypes compared with those carrying T allele, thus they might have less protective effects on normal tissues, and increased cancer risks. However, when stratified by HWE status of controls, we found that significantly elevated risks were observed in HWE inconsistent studies, but not in HWE consistent studies. Thus there may be possibility that the presence of the C allele is in linkage disequilibrium with another mutation located outside the coding region in the *XRCC6* gene, which may be important for the *Ku70* expression.

The rs132770 polymorphism locates closer than rs5751129 polymorphism to the translation starting point in the *XRCC6* promoter. Our meta-analysis indicating that rs132770 polymorphism might affect RCC risk in a different way from in other cancers, the RCC pathogenesis mechanism might be distinguished from other cancers when rs132770 polymorphism involved. The results were consistent with the finding that the A allele of the rs132770 polymorphism could increase the expression levels of the Ku70 mRNA in normal tissue of RCC patients.^21^ Xu^17^ also collected 3 studies but failed to find any association. The negative results in Xu study might be due to the limited studies.

It is interesting that the rs132793 polymorphism was found to play opposite roles in different cancers: it might decrease breast cancer risk, whereas increase cancer risk in “other cancers”, indicating that the rs132793 may play opposite roles in breast cancer contrast to other cancers. The rs132793 locates in the sequences downstream of stop code of *XRCC6* gene, and until recently, the role of rs132793 in tumorigenesis is unclear. We hypothesized that the rs132793 polymorphism might influence breast cancer risk via some mechanisms different from other cancers. Functional studies should be carried out to explore the mechanisms involving rs132793 polymorphism in tumorigenesis in the future. The different ethnicities of the study population might influence the genetic effect of the rs132793 polymorphism on cancer susceptibility; anyhow, it seems not substantial in our study.

Our results implied that rs132774 polymorphism have no association with cancer risk in Chinese population. To validate the results, more population-based studies in other populations should be carried out in the future.

There was heterogeneity among studies in rs2267437, rs5751129, and rs132793 polymorphisms. “Source of control” and “ethnicity”, “cancer type” might explain 100% of the τ^2^ in the rs5751129 and rs132793 polymorphism, respectively. However, the combination of “ethnicities”, “source of control”, and “sample size” could explain only 17.6% of the τ^2^ in heterozygote model in rs2267437 polymorphism, implying that there may be other reasons accounting for the heterogeneity in the rs2267437 polymorphism. Publication bias was found in the rs5751129 polymorphism and trim and fill method could reduce the influence of publication bias in all models except recessive and homozygous model, implying that more cautions should be paid when elucidate the role of the rs5751129 polymorphism in cancer susceptibility. Anyhow, sensitivity analysis proved that the results of this meta-analysis were statistically reliable. Therefore, a methodologically preferable design, such as using population-based controls, is crucial to avoid selection bias and heterogeneity.

The limitations of our meta-analysis should also be discussed. First, the non-English articles were excluded in our study, which thus may bias the results of our results. Second, some low-quality studies with deviation from HWE in the control group were included in our meta-analysis. Subgroup analysis were carried out by HWE status of controls in rs5751129 polymorphism and rs132770 polymorphism, and the opposite results were found in rs5751129 polymorphism between HWE consistent/inconsistent groups, implying that low-quality studies might influence the results in rs5751129 polymorphism. Third, the number of included studies was relatively small in some subgroups, thus the specific results should be interpreted with caution.

In conclusion, this meta-analysis showed some evidence of the *XRCC6* SNP polymorphisms and cancer risk, supporting the existence of association between *XRCC6* polymorphisms and cancer risks in different ethnicities and cancer types. The rs2267437 polymorphism was found to be associated with a significant increase in risks of overall cancers, breast cancer, RCC and HCC, and it might increase the cancer risk in Asian population. The rs5751129 polymorphism might increase the cancer risk in overall cancers, and the rs132770 polymorphism might be associated with the increased cancer risk in RCC. Furthermore, the rs132793 polymorphism could decrease breast cancer risk and increase risks in “other cancers”. However, there was no obvious association between *XRCC6* polymorphisms and risks of some “other cancer”. This may be due to the limited studies included or limited sample size in “other cancers”. Therefore, more studies with large sample are required to further confirm the results in the future. Functional studies are also needed to elucidate the roles of *XRCC6* promoter polymorphisms in cancer pathogenesis. Moreover, gene–environment and gene–gene interaction analyses, as well as haplotype analysis should be carried out to clarify the role of the *XRCC6* genes in cancer. Our studies may perhaps supplement for the disease monitoring of cancers in the future, and additional studies to determine the exact molecular mechanism might provide us with interventions to protect the susceptible subgroups.
